# Computer-Based Mechanobiological Fracture Healing Model Predicts Non-Union of Surgically Treated Diaphyseal Femur Fractures

**DOI:** 10.3390/jcm12103461

**Published:** 2023-05-14

**Authors:** Christina Degenhart, Lucas Engelhardt, Frank Niemeyer, Felix Erne, Benedikt Braun, Florian Gebhard, Konrad Schütze

**Affiliations:** 1 Department of Trauma-, Hand-, and Reconstructive Surgery, Ulm University, Albert-Einstein-Allee 23, 89081 Ulm, Germany; christina.degenhart@uniklinik-ulm.de (C.D.);; 2 OSORA—Medical Fracture Analytics, Helmholtzstr. 20, 89081 Ulm, Germany; lucas.engelhardt@osora.eu (L.E.);; 3 Department of Trauma and Reconstructive Surgery, Eberhard-Karls-University Tuebingen, BG Unfallklinik, 72076 Tuebingen, Germany

**Keywords:** non-union, delayed union, computer-based simulation, diaphyseal femur fracture, fracture healing simulation, mechanobiological healing model, numerical simulation model, healing prediction

## Abstract

As non-unions are still common, a predictive assessment of healing complications could enable immediate intervention before negative impacts for the patient occur. The aim of this pilot study was to predict consolidation with the help of a numerical simulation model. A total of 32 simulations of patients with closed diaphyseal femoral shaft fractures treated by intramedullary nailing (PFNA long, FRN, LFN, and DePuy Synthes) were performed by creating 3D volume models based on biplanar postoperative radiographs. An established fracture healing model, which describes the changes in tissue distribution at the fracture site, was used to predict the individual healing process based on the surgical treatment performed and full weight bearing. The assumed consolidation as well as the bridging dates were retrospectively correlated with the clinical and radiological healing processes. The simulation correctly predicted 23 uncomplicated healing fractures. Three patients showed healing potential according to the simulation, but clinically turned out to be non-unions. Four out of six non-unions were correctly detected as non-unions by the simulation, and two simulations were wrongfully diagnosed as non-unions. Further adjustments of the simulation algorithm for human fracture healing and a larger cohort are necessary. However, these first results show a promising approach towards an individualized prognosis of fracture healing based on biomechanical factors.

## 1. Introduction

The treatment of femoral non-unions is a huge challenge for every surgeon. Non-unions after reamed intramedullary nailing are rare. Nevertheless, their existence causes severe pain, a prolonged disability, poor quality of life, and poor mental health, as well as high costs due to an increased re-hospitalization rate [1,2,3]. The rate of delayed unions or non-unions in femoral shaft fractures varies from study to study. In the literature, figures from 1.6 to 12% after treatment with intramedullary nailing are reported [4,5]. In a recently published study, Mills et al. evaluated the overall risk of non-union at 1.9% [6].

**Figure 1:**
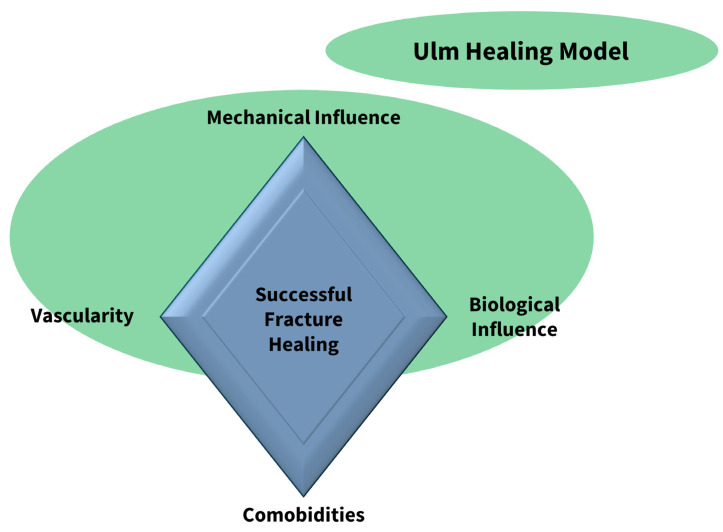
Fracture Diamond. Based on the fracture diamond model established by Giannoudis et al. [9], the Ulm Healing Model is able to integrate mechanical and biological influences, as well as vascularity, to a certain point. Concomitant diseases cannot be integrated at this moment.

Reasons for non-unions are, most commonly, biomechanical instability and infections, as well as patient-based criteria such as comorbidities or nicotine abuse. Especially instabilities caused by a too small nail diameter, fracture dehiscence, and misalignment are relevant [7,8]. According to the fracture diamond model of Giannoudis et al., bone healing is mainly influenced or compromised by biological, vascular, mechanical, and patient-specific factors [9] (Figure 1).

Despite this knowledge, the correct prognosis of non-union is difficult and mainly depends on the surgeon’s experience. Additionally, the exact timing of the revision is hard to identify.

Multiple clinical-radiological scores have been established to identify possible non-unions, especially tibial (NURD, LEG-NUI, etc.) [10] and subtrochanteric non-unions [11]. No score exists for diaphyseal femur fractures. The NUSS score, created by Calori et al., can be used as a multifactorial approach to the estimation of non-union risk, taking bone stock, surrounding soft tissue, and patient health into consideration. Fifteen factors influencing non-union are evaluated, and the scores are added and multiplied by two. A maximum of 100 points can be reached, indicating maximum risk for impaired healing. Using the ladder strategy, a certain treatment is recommended [12]. The score has been validated by several authors [13,14]. The main point of criticism is the underestimation of necessary treatment due to biological factors [15]. All scores are based on patient comorbidities and surgical factors, as well as on the evaluation of postoperative and follow-up X-rays. A further development might be to use computational methods to take biomechanical aspects as well as vascularity into consideration.

In addition to these purely radiological rating systems, it has been proposed to use measures characterizing mechanical stability as a predictor for healing success instead. For instance, Perren defined the interfragmentary strain (IFS) for characterizing the biomechanical environment at the fracture site [16]. Using simple global measures, such as IFS, as a predictor for healing success in real-world cases, however, is challenged not only by the difficulty of even defining them for complex fractures with varying fracture gap sizes and complex physiological motion, but also by the fact that a single value can hardly sufficiently describe the complex nature of the actual strain field.

Over the course of the last three decades, multiple mechano-regulation hypotheses of tissue differentiation have been proposed and implemented in numerical simulation models of bone remodeling and fracture healing that consider the full strain field in order to predict local tissue differentiation [17,18]. One of these models, the Ulm tissue-level bone healing model [19,20], based on the biomechanical thesis by Pauwels [21] with further refinements by Claes and Heigele [22], unifies both remodeling and fracture healing in a single model and is able to integrate patient-specific information and all current complex information on bone healing. Local distortional and dilatational strains have been identified as the main mechanical stimuli for tissue differentiation and remodeling (woven and lamellar bone, cartilage, and connective tissue) [20]. Aside from mechanical stimuli, the fuzzy logic rules take local tissue composition as well as vascularization into account. Through iterative numerical procedures, the tissue differentiation processes of fracture healing are simulated [23]. After having been corroborated by in vivo animal experiments, the next step will be using the model on a human collective under clinical conditions [24].

The aim of this study was to establish a simulation-based method that allows for the identification of patients at risk for developing non-unions due to biomechanical problems by predicting the individual healing process based on postoperative data. If successful, this method would enable improved individualized care by providing surgeons with additional data to base their clinical decisions on. Due to the limited nature of the available retrospective data, we focus on the model’s capability of predicting the outcome as “union”/“non-union”. Consequently, predictions of the exact tissue differentiation process or the callus’ size, etc., could not be validated within the context of this study and should therefore be considered purely hypothetical.

Our hypothesis proposes that our computer-based simulation correctly predicts healing, or non-union, of femoral shaft fractures after surgical treatment with intramedullary nailing. The bridging dates predicted by the simulation are then correlated retrospectively with the clinical and radiological healing processes of the patients, and the accuracy of the prognosis is thus evaluated.

## 2. Materials and Methods

### 2.1. Data Collection and Simulation Procesdure

In this feasibility study, 32 cases were included. Six patients had developed non-union in the clinical course. All patients have been treated with intramedullary nailing between 2010 and 2020 in a level one trauma center. The study has been approved by the Ethical Review Committee of the University of Ulm (136/22—FSt/Sta).

All fractures were closed femoral shaft fractures that have been rated A1–C2 according to the AO classification and have been treated with intramedullary nailing. Patients between the ages of 16 and 65 were included. Exclusion criteria were other treatment than nail, pathological fractures, subtrochanteric fractures, further fractures of the lower extremities impairing full weight-bearing, comorbidities, slowing down fracture healing, and fracture-related infections. Basic information such as age, height, body weight, and additional information such as comorbidities were extracted from patient charts.

After anonymizing the data and creating the 3D models based on the post-OP X-ray data, the simulations were performed on the “bw-UniCluster 2.0” HPC cluster (High Performance Computing and Large-Scale Scientific Data Management Baden-Württemberg).

### 2.2. Geometry Reconstruction

Simulating the healing process requires a three-dimensional model of the patient’s anatomy. Based on the postoperative biplanar X-rays of each patient, a standardized femur model was adapted [25]. This geometry also included surfaces where the muscles are attached to the femur. First, the femur’s outer proportions were scaled to match those of the X-rays from both views. In a second step, the cortical thickness was adapted to match the X-rays. In a third step, the approximate fracture geometry was recreated at the location visible in the images. Last, we aligned the bone segments according to the repositioning and retention conducted by the surgeon to reproduce a possible malalignment of the fracture.

### 2.3. Intramedullary Nail

Using Open CASCADE Technology in Pygmsh 7.1.17, we reverse engineered parametric models of the intramedullary nails (LFN and PFNA long, FRN by De Puy Synthes) that were used to treat the patients. For each patient, we chose the corresponding sized implant according to the medical report of each patient and placed the simulated nail within the medullary canal according to the real configuration and alignment as visible in the post-surgical X-rays. Furthermore, screws (dynamic or locking) were added according to the image data.

### 2.4. Finite Element Dicretization

With the aid of ANSYS Mechanical (Release 2020 R1; ANSYS, Inc., Canonsburg, PA, USA), the resulting geometries were discretized in quadratic tetrahedral finite elements.

Mesh convergence analysis, as suggested by Viceconti et al. [26], was carried out to identify a suitable fine meshing of the entire domain. Resulting models have numbers ranging from 890,436 to 1,647,232 elements, depending on the geometry complexity.

### 2.5. Load and Boundary Conditions

All patients were allowed to bear full weight after surgery. Because there was no information about the actual weight-bearing, the maximum load occurring during the normal gait cycle was assumed to be full weight-bearing. Therefore, the muscle and joint loading conditions, as stated by Heller et al., 2005 [27], were used, which allows to express loading in terms of percentage of patients’ bodyweight. Heller et al. determined a simplified muscle-loading scheme for walking that maintains physiological hip-joint loading. Specifically, body weight-scaled contributions from the gluteus medius, minimus, and maximus, the vastus lateralis, and the proximal and distal parts of the tensor fascia latae were simulated. Muscle loads were applied to the geometric femur model using the muscle insertion surfaces defined by Viceconti et al. [25].

### 2.6. Fracture Healing Simulation

**Figure 2:**
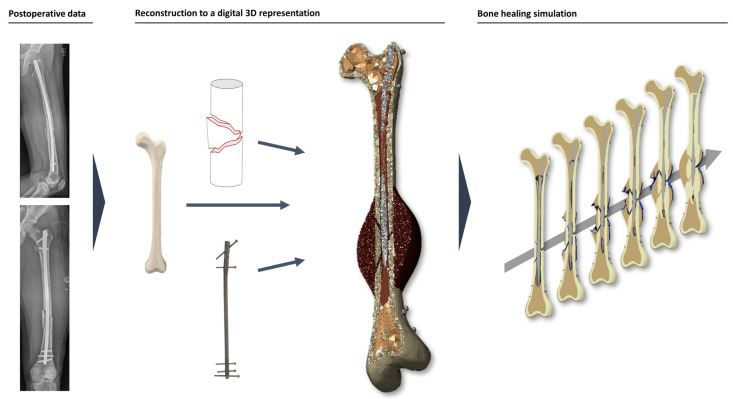
Simulation process. A 3D representation of the patient’s femur is reconstructed by using postoperative biplanar X-rays. Patient geometry, nail parameters, and the actual fracture site are inserted into the simulation. Based on the patient’s weight and height, the bone healing simulation calculates the formation of calluses and, therefore, healing vs. non-union.

For simulating the healing process, we used the latest version of the Ulm bone healing model, previously described in detail by Engelhardt et al., 2021, and Niemeyer et al., 2018 [23,28], without any further modification. Briefly, the model predicts the change in tissue composition (fibrous connective tissue, fibrocartilage, woven bone, lamellar bone) depending on mechanical and biological stimuli (distortion and dilatational strain, local and adjacent tissue composition, vascularity) at each point of the healing region. The amount and distribution of mechanical stimuli depend on the assumed load case and mechanical boundary conditions, as well as the material properties and spatial distribution of the tissue types (see next paragraph). By iteratively computing the current strains via FEM (Finite Element Method, ANSYS Mechanical (Release 2020 R1; ANSYS, Inc., Canonsburg, PA, USA)) and subsequently updating the tissue composition of the healing region, the model predicts tissue differentiation over time, including bone formation and remodeling, from which the time of consolidation can be derived (Figure 2).

## 3. Evaluation

### 3.1. Clinical Results

The time point of radiological bridging of three cortices was defined as the clinical consolidation point. Non-healing was extracted from the postoperative documentation (combined clinical and radiological results). These results were compared retrospectively with the simulated data using the RUST score for assessment of the radiographic healing [25,26].

We adapted the RUST concept for scoring callus formation in tibia fractures to compare clinical and virtual data objectively. The RUST- or mRUST-score has previously been used and evaluated for femur fractures [29,30]. Callus formation is rated on a continuous scale from 4 (no healing) to 16 (maximal healing) points. In the presence of a callus without a visible fracture line, 4 points are given: bridging callus with a visible fracture line (3 points), non-bridging callus (2 points), and no callus (1 point). Each cortex is evaluated separately [31]. A value of 9 was defined as consolidated. Clinical non-union was diagnosed by the documented prolonged pain at the fracture site and a mRUST score below 9 on either X-ray or CT.

### 3.2. Simulation Results

The primary result of the simulation is the spatio-temporal distribution of tissue concentrations. To compare these results to the clinical outcomes, the simulation results were evaluated qualitatively and quantitatively. A qualitative assessment was performed by visually comparing clinical X-rays with virtual X-rays, which were rendered from the predicted tissue distribution at the time-points corresponding to the available clinical X-rays. Furthermore, a quantitative analysis was performed. The bridging time points for all four quadrants (medial, lateral, anterior, and posterior) of the femur were computed by searching for a continuous connection of lamellar bone (≥70%) from the top-most to the bottom-most point via a path search algorithm. If fewer than 3 quadrants within 280 simulated days (9 months) were identified as bridged, the case was categorized as a non-union.

## 4. Results

All patients were treated between 2010 and 2020 in a level 1 trauma center in Germany. Applying the above-mentioned inclusion and exclusion criteria, we identified 32 suitable cases. We decided to focus on osteosynthesis with LFN, PFNA long, and FRN (DePuy, Synthes, Johnson & Johnson Services, New Brunswick, NJ, USA) and excluded 5 patients who were treated with nails from other manufacturers (AFN, Trigen). One patient could not be simulated due to meshing problems, and one patient had to be excluded because of cerclages and one due to insufficient X-ray data. Simulating cerclages is per se possible, but the positioning of the cerclages is dependent on the fracture configuration, so there is no standardization for implantation. Therefore, including cerclages would have increased the amount of time needed for simulation significantly.

In summary, 27 patients (7 females, 20 males), aged 16–60 with a mean age of 30.4 ± 14.5 years could be evaluated, including 10 right and 17 left femora. The mean patient height was 173.4 ± 12.8 cm, and the mean patient weight was 80.4 ± 18.3 kg, with a mean BMI of 26.5 ± 5.4 kg/m^2^. A total of 17 patients fractured their femur during high-velocity traffic accidents; 1 trauma mechanism was unknown; the other fractures occurred due to falls.

**Figure 3:**
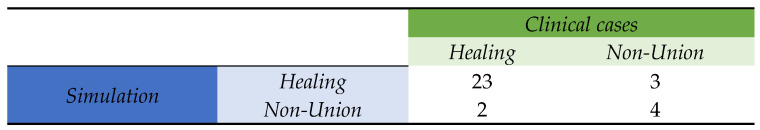
Confusion matrix comparing clinical to predicted outcomes.

In total, 32 simulations were performed. The 29 LFN, 1 FRN, and 2 PFNA were used for osteosynthesis. Clinically, in 25 cases, healing took place without problems; in 7 cases, non-union occurred, including the 5 non-union patients who were simulated again, according to the new osteosynthesis, after revision was performed. After revision, 4 patients healed without further complications; 1 patient developed non-union again (Figure 3).

**Figure 4:**
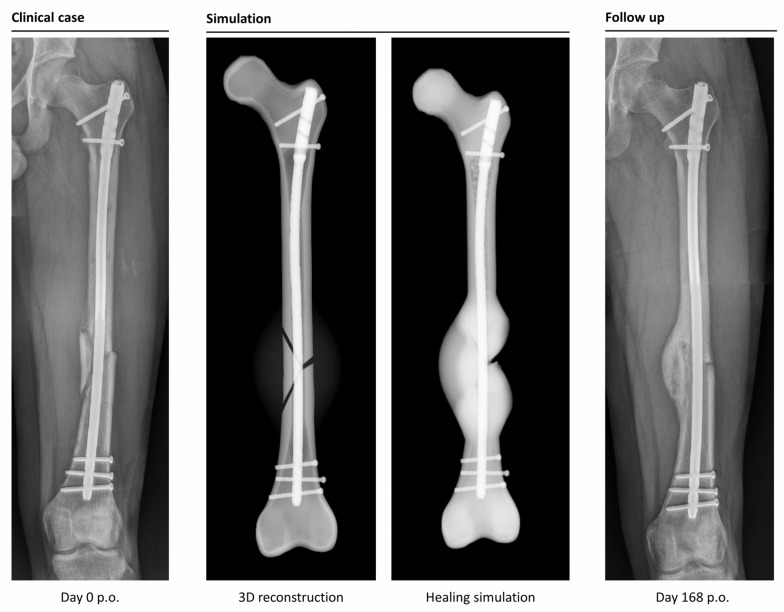
Example of a correctly simulated, healing fracture. The postoperative X-ray (picture 1, left) is used to build a 3D reconstruction (picture 2). The simulation correctly predicts uncomplicated healing (picture 3). A retrospective comparison to a follow-up X-ray on day 168 after surgery shows similar callus formation and progressing consolidation (picture 4, right).

**Figure 5:**
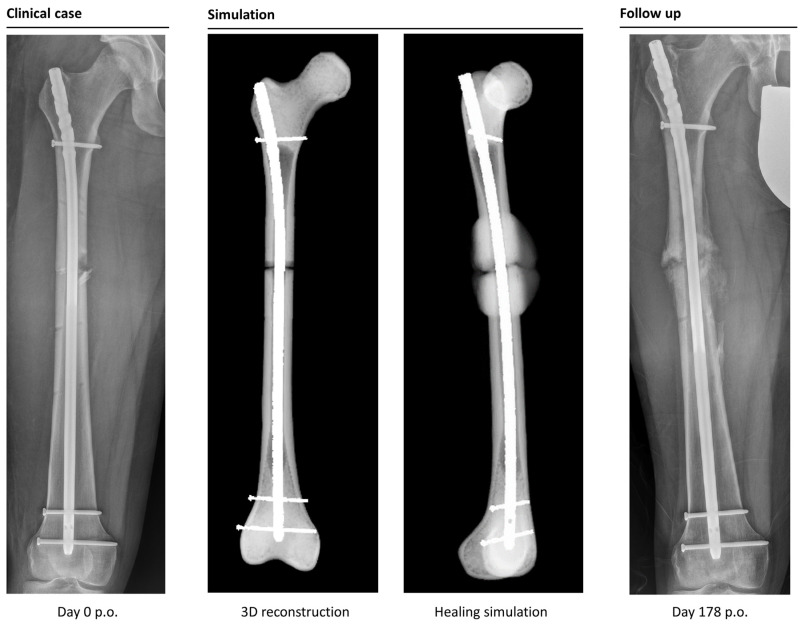
Example of a correctly simulated non-union fracture. The postoperative X-ray (picture 1) is used to build a 3D reconstruction (picture 2). The simulation correctly predicts a biomechanically induced non-union (picture 3). A retrospective comparison to a follow-up X-ray on day 178 after surgery shows similar callus formation and no healing, resulting in a consecutive revision surgery (picture 4).

The simulation classified 26 cases as “healing”, while it categorized 6 cases as non-unions. In retrospective comparison with the clinical outcomes, the simulation correctly predicted 23 uncomplicated healing fractures. Three patients would have been expected to heal according to the simulation, but clinically turned out as non-unions (false negatives). Four out of six predicted non-unions were correctly recognized by the simulation, but two cases were wrongfully diagnosed as developing non-unions (false positives). This means the simulation correctly predicted the right outcome in 85% of the cases (Figure 4 and Figure 5).

**Figure 6:**
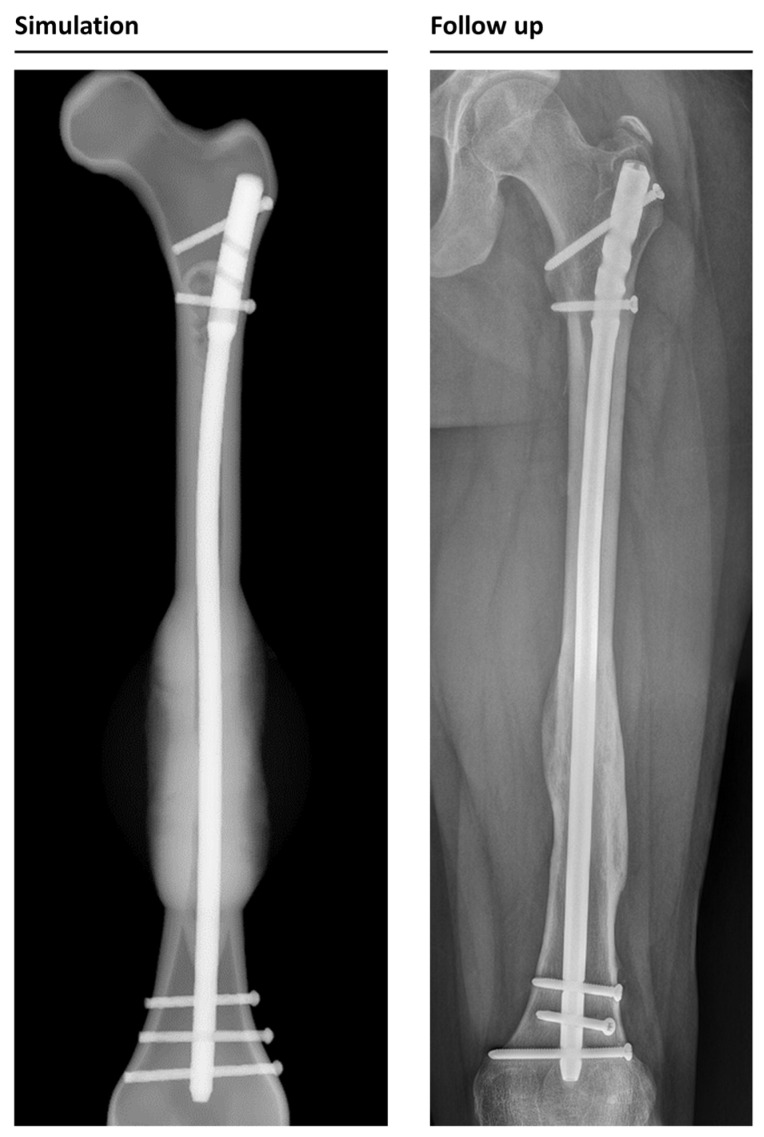
Callus formation in the simulation and follow-up X-ray data in anterior-posterior view. Follow-up was taken 31 weeks after surgery; simulation shows the same timeframe.

To compare virtual and clinical outcomes, the mRUST score was evaluated for all cases. “Healing” was defined as the first time an mRUST score over 9 was reached, which means a bridging of at least 3 cortices. The mean mRUST score was 9.5, whereas the time of follow-up X-rays varied in such a range that no direct comparison is possible (Figure 6).

**Figure 7:**
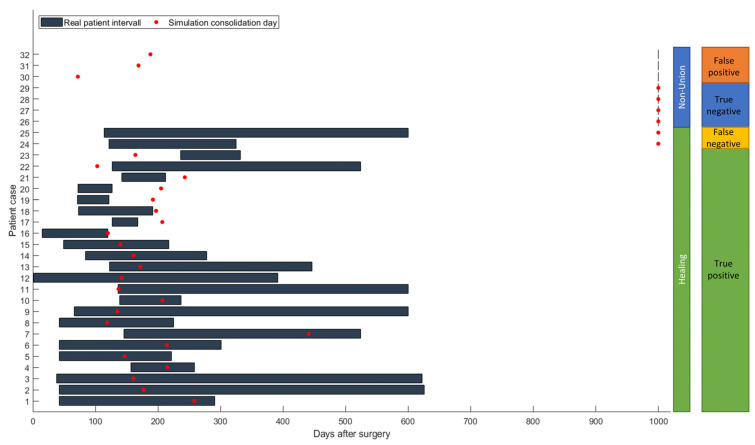
Comparison of consolidation; True positive—consolidation in simulation and reality; false negative—non-union in simulation but clinical consolidation; true negative—non-union in simulation and clinic; false positive—consolidation in simulation but not in reality.

For the 23 cases where the simulation correctly predicted consolidation, according to the simulation, average consolidation took place 189.5 ± 61.4 days after surgery. Due to the retrospective comparison, no definitive clinical healing time can be calculated, and therefore a direct comparison is not possible. Clinical consolidation took place from days 120 to 626 after surgery, so all healed patients developed an mRUST score of at least 9 during this time (see Figure 7).

A further aim of the study was the comparison of the bridging times predicted by the simulation with the clinically observed ones. For 16 of these 23 cases (P01–P16), the predicted healing time point was compatible with the clinical consolidation time range that could be deduced from the retrospective radiographic data. In five cases (P17–P23), clinical healing was faster than the simulated healing, and in two cases, it was slower.

## 5. Discussion

In this study, to the best of our knowledge, the Ulm fracture-healing model was used for the first time in a clinical context to predict non-unions. After further adjustment of the calculation on human bones, this system could be used as an individualized approach to early detection of delayed or non-union. Clinical intervention could be derived, or alternatively delayed, in cases of retarded but late healing.

Clinical consolidation was correctly predicted in 23 cases; 3 cases were wrongfully diagnosed as healing in the simulation but resulted in non-union clinically. In the retrospective analysis of the cases, no general, documented clinical deviation could be detected. In these cases, solely relying on the simulation would have led to an overly optimistic estimation of the healing chances. Aside from shortcomings of the simulation model, possible extrinsic reasons for non-detection could be undocumented patient-specific factors impairing fracture healing, e.g., unreported nicotine consumption or incompliance concerning weight-bearing. Patient compliance concerning weight-bearing as well as physiotherapeutic exercise contributes to healing. This compliance has not been observed, and actual loading can only be estimated by the simulation.

Furthermore, a need for further adaptation and improvement of the model is under discussion. For instance, the simulation assumes the same muscle insertion points for all individuals as well as the same muscle force distribution. This may also contribute to inexact results. For example, musculoskeletal forces are calculated indirectly, and direct measurement in vivo is not possible. Additionally, the initial soft tissue distribution and vascularization can only be estimated and approximately calculated.

A further modification of the model for compromised bones (e.g., poor bone quality caused by osteoporosis) might be necessary in order to predict healing in severe cases of osteoporosis. After speeding up the process of simulation, large numbers of patients with different conditions impairing fracture consolidation could be analyzed to take co-morbidities into consideration. At this time, further approaches are being made by other scientific groups to adjust fracture healing algorithms, e.g., in humans with diabetes [18]. Integration of these findings into the simulation is possible in principle.

Concerning the six cases where the simulation predicted a non-union, four matched the clinical outcome. Taking a closer look at the two falsely detected non-union cases, a possible reason is the fracture configuration. Patients 24 and 25 both showed fractures with high distortional strain and, therefore, prolonged vascularization of the formed callus in the simulation. In both cases, the clinical course showed a rather large callus formation but a normal consolidation time. A possible explanation may be, on the one hand, an overestimated loading and, on the other hand, underestimated osteosynthesis stability. A second possible explanation might be that the model allows a too small window of distortional strain. As published by Dailey et al., the tissue destruction cutoff for distortional strain seems to have to be increased from the previously published cutoff of 0.17 to an upper limit of 1.0 to accurately depict the actual influence of distortional strains [18]. This issue might be an artifact of deriving the differentiation rules from animal models. Further adjustment may be necessary to adapt the model to human bone healing.

The average time for the bridging of three cortices according to the simulation was 184 days, which is longer than the clinically expected 6 to 12 weeks. Yet, comparing the radiologically determined healing intervals to the healing time points predicted by the simulation model, in 16 out of 23 cases, the predicted consolidation speed was compatible with the clinical data. Due to the retrospective character of the study, X-ray intervals varied widely between cases, and further narrowing down the time of actual consolidation was not possible. In six cases, consolidation took place earlier than predicted by the simulation. We concluded that this may also indicate the necessity of adapting the calculation to human healing potential. Narrowing down the time of radiologic healing may help to further evaluate the exactness of the simulation. Due to the retrospective character of this study, this has proven difficult due to the lack of follow-up examinations or the large amounts of time between X-rays. This could be further evaluated by a prospective study and an extension of the patient collective.

There is a lack of certain tools to assess fracture healing. Currently, the evaluation of the progress of fracture healing is mainly based on clinical and radiological findings as well as on surgeons’ experience. Especially in the so called “forgotten phase” (the time 6 to 9 months after surgery), in which delayed union cannot be detected clinically or radiologically, surgical intervention is stalled. This leads to decelerated treatment [32].

Using radiology as the main tool to detect non-union is only possible during healing, and a reliable prediction of consolidation is not possible. X-rays to assess fracture healing are a cheap and quick method, but nevertheless, various studies show inaccurate and inconclusive findings concerning the diagnosis of non-union, as well as a strong dependence on the viewer’s experience. Scoring systems such as RUSH and RUST scores have been established to create a comparable and reliable score for radiological assessment of fracture healing, but their accuracy is still under evaluation [12]. CT offers high sensitivity to detect non-unions but low specificity. Due to cost, radiation dose, and metal artifacts, diminishing the accuracy, CT diagnostics is not a widespread way for early detection of non-union [13]. Ultrasound, as used by Moed et al. in tibial fractures, is not tested for femur fractures [14]. DCE-MRI perfusion analysis after revision surgery was shown to successfully predict the postoperative outcome but is not common and expensive [33].

Currently, scientific approaches to measuring fracture healing include, e.g., implanted magnetoelastic wireless electronic devices, analysis of vibrations through the bone, and fluidic X-ray-sensitive sensors attached to osteosynthesis material. None of these methods is practical or fit for widespread clinical use, and they have not yet been adequately tested [15].

As serological markers for fracture healing, bone-specific alkaline phosphatase (BALP), procollagen type-III N-terminal propeptide (PIIINP), procollagen type-I C-terminal propeptide (PICP), and osteocalcin (OC) are often discussed. Further evaluation and interpretation of these findings in the context of non-union development is under current examination [16,17].

At this point, none of the above-mentioned conventional and experimental tools can reliably predict non-unions early enough. Using big data may be a solution to the problem of early detection.

This study was performed as a feasibility approach to use the Ulm fracture healing model to simulate consolidation. The model has been used for different scientific questions but has never been used on clinical human data [28,34,35]. Ideally, we would like to obtain detailed data about the temporal evolution of the different tissue types at each point of the fracture site in order to enable a quantitative comparison of the primary simulation output with reality. Unfortunately, obtaining such data, retrospectively, is currently not feasible. We therefore focused the discussion on derived predictions, such as classifying union/non-union cases, that can readily be compared to clinical data. One obvious limitation of this approach is that the predicted evolution of tissue distribution, callus growth, and calcification remains hypothetical. Perhaps more serious, however, is the fact that it does not allow us to infer why the model might have failed in a specific case, making it virtually impossible to use the outcome of this study to improve the model.

While it might be tempting to replace complex tissue differentiation models with their many unknown parameters with a simpler model that is easier to validate, it is not a straightforward task to define such an indicator. For instance, when applying Perren’s idea of IFS to the data set at hand, we did not find any significant difference between the groups of healing/non-union cases with respect to initial IFS. Again, given enough data, it is conceivable to identify more complex patterns of the initial biomechanical condition (e.g., via machine learning) that are indeed useful predictors of the healing outcome, but that comes at the expense of replacing a simulation model that is built on first principles and could—at least in theory—offer some explanation for its predictions with an opaque black box model.

Tibial non-unions are more often reported than femoral non-unions, but they are mostly accompanied by or caused by extensive soft tissue damage. To focus on the biomechanical aspect of the model, we decided to focus on femur fractures before including tibial fractures. Additionally, the medical relevance of femoral non-unions is given by the relevant impact on patients’ health caused by misdiagnosis or delayed diagnosis of non-unions concerning femur fractures. Geometric variation and study data were sufficient to use femora as a starting point.

A limitation of this study is the use of biplanar X-rays to assess the initial fracture geometry. Complicated or multi-fragment fractures can only be reconstructed approximatively. To recreate the real geometry, CT data would be necessary. Yet, CT imaging is not needed for definitive treatment and therefore only exists in rare cases or to detect delayed union.

Furthermore, given the limited amount of information we had available about each case and given that we explicitly excluded patients with comorbidities that are known to influence fracture healing specifically, such as patients with osteoporosis or smokers, we assumed the same “biology” (i.e., tissue remodeling rates, sensitivity to stimuli) for all patients.

Regarding patient-specific loading, we assumed full weight-bearing for all cases throughout the healing process. It is known that patients are typically not able to follow conventional loading recommendations [36]. By treating all cases equally, we avoid introducing artificial bias in individual cases. Assuming that biomechanically induced non-unions in the case of fractures treated with an intramedullary nail tend to be a consequence of insufficient stability, we implicitly increase the sensitivity of the model for detecting non-union cases, with the trade-off of an increased false-positive rate.

After further re-evaluation and testing of the model on a larger collective, we are positive that it is possible to assess the risk for non-unions using our simulation. Further work must be conducted to accelerate the speed of calculation to make the model usable for clinical work as a diagnostic tool for personalized treatment. One possibility of use might be to evaluate the risk of non-union directly after surgery to prevent long-term disability and pain for the patient and evaluate the need for immediate revision. To achieve this goal, further studies with larger sample sizes need to be conducted in order to tune the model further to human differentiation rates, as it was originally established and calibrated using in vivo data from sheep and mouse models.

Within the hospital workflow, another benefit, apart from detecting non-unions early on, might be in planning further examinations and giving recommendations for weight bearing. For patients with a positive prognosis for successful healing, it might be possible to extend the intervals of routine control, reducing radiation exposure. Finally, an intraoperative risk assessment would enable surgeons to adjust the treatment plan during the surgery based on real-time information, thereby increasing the precision and effectiveness of the procedure. Furthermore, visualizing the healing process may also help increase patient compliance.

As a further step, it might be possible to develop a tool for preoperative estimation of healing potential depending on different osteosynthesis options, e.g., nail diameter, dynamic vs. static locking, or decision nail vs. plate, even before the surgical intervention takes place.

## 6. Conclusions

This is the first time the Ulm fracture-healing model has been applied to clinical data. The results demonstrate that it is possible to simulate clinical fracture healing based on a mechanical and biological basis. This is the first step towards an individualized approach to healing assessment. Our promising data show that predicting fracture healing by computer simulation is possible under certain circumstances and may be, after further re-evaluation and process improvement, a future opportunity to assess the biomechanical healing potential, possibly even under consideration of co-morbidities. This may allow early detection and addressing of non-unions and therefore reduce the risk of prolonged suffering and a delayed return to work.

## Data Availability

The anonymized created data can be requested after consultation with the authors via the above mentioned corresponding email address.

## Abbreviations

PFNA	Proximal Femoral Nail Antirotation
LFN	Lateral Femoral Nail
(m)RUST	(Modified) Radiographic Union Scale for Tibia Fractures
CAD	Computer Aided Design
FEM	Finite Element Method
AFN	Antegrade Femoral Nail
